# A Systematic Study of Popular Software Packages and AI/ML Models for Calibrating In Situ Air Quality Data: An Example with Purple Air Sensors

**DOI:** 10.3390/s25041028

**Published:** 2025-02-09

**Authors:** Seren Smith, Theodore Trefonides, Anusha Srirenganathan Malarvizhi, Shyra LaGarde, Jiakang Liu, Xiaoguo Jia, Zifu Wang, Jacob Cain, Thomas Huang, Mohammad Pourhomayoun, Grace Llewellyn, Wai Phyo, Sina Hasheminassab, Joe Roberts, Kevin Marlis, Daniel Q. Duffy, Chaowei Yang

**Affiliations:** 1 NSF Spatiotemporal Innovation Center, George Mason University, 4400 University Dr., Fairfax, VA 22030, USA; jsmit92@gmu.edu (S.S.); ttrefoni@gmu.edu (T.T.); asrireng@gmu.edu (A.S.M.); slagarde@gmu.edu (S.L.); jliu22@gmu.edu (J.L.); xjia5@gmu.edu (X.J.); zwang31@gmu.edu (Z.W.); jcain8@gmu.edu (J.C.); 2 NASA Jet Propulsion Laboratory, 4800 Oak Grove Dr., Pasadena, CA 91011, USA; thomas.huang@jpl.nasa.gov (T.H.); grace.llewellyn@jpl.nasa.gov (G.L.); wai.phyo@jpl.nasa.gov (W.P.); sina.hasheminassab@jpl.nasa.gov (S.H.); joe.t.roberts@nasa.gov (J.R.); kevin.marlis@jpl.nasa.gov (K.M.); 3 Department of Computer Science, California State University, 1250 Bellflower Blvd, Long Beach, CA 90840, USA; mpourho@calstatela.edu; 4 NASA Goddard Space Flight Center, Greenbelt, MD 220771, USA; daniel.q.duffy@nasa.gov

**Keywords:** sensors, air quality, calibration, particulate matter, AI/ML, model accuracy, environment, sensor calibration

## Abstract

Accurate air pollution monitoring is critical to understand and mitigate the impacts of air pollution on human health and ecosystems. Due to the limited number and geographical coverage of advanced, highly accurate sensors monitoring air pollutants, many low-cost and low-accuracy sensors have been deployed. Calibrating low-cost sensors is essential to fill the geographical gap in sensor coverage. We systematically examined how different machine learning (ML) models and open-source packages could help improve the accuracy of particulate matter (PM) 2.5 data collected by Purple Air sensors. Eleven ML models and five packages were examined. This systematic study found that both models and packages impacted accuracy, while the random training/testing split ratio (e.g., 80/20 vs. 70/30) had minimal impact (0.745% difference for R^2^). Long Short-Term Memory (LSTM) models trained in RStudio and TensorFlow excelled, with high R^2^ scores of 0.856 and 0.857 and low Root Mean Squared Errors (RMSEs) of 4.25 µg/m^3^ and 4.26 µg/m^3^, respectively. However, LSTM models may be too slow (1.5 h) or computation-intensive for applications with fast response requirements. Tree-boosted models including XGBoost (0.7612, 5.377 µg/m^3^) in RStudio and Random Forest (RF) (0.7632, 5.366 µg/m^3^) in TensorFlow offered good performance with shorter training times (<1 min) and may be suitable for such applications. These findings suggest that AI/ML models, particularly LSTM models, can effectively calibrate low-cost sensors to produce precise, localized air quality data. This research is among the most comprehensive studies on AI/ML for air pollutant calibration. We also discussed limitations, applicability to other sensors, and the explanations for good model performances. This research can be adapted to enhance air quality monitoring for public health risk assessments, support broader environmental health initiatives, and inform policy decisions.

## 1. Introduction

Climate change, urbanization, fossil fuel energy consumption, and other factors have exacerbated air pollution and related public health issues [1,2]. Effective Air Quality (AQ) monitoring is vital for safeguarding public health, especially in densely populated urban areas where pollution levels are often higher. Traditional high-cost, high-maintenance AQ monitoring systems, while accurate, are often limited in geographic coverage and flexibility, making comprehensive AQ monitoring and surveillance challenging. Recent advancements in sensor technology and manufacturing have seen a rise in the application of low-cost sensors (LCSs), which offer broader geographic coverage.

The advent of LCSs presents a transformative challenge and opportunity to enhance AQ monitoring. Purple Air (PA) sensors, one type of low-cost sensor, have gained prominence due to their affordability, ease of deployment, and timely readings [3]. However, the practical utilization of these sensors is significantly compromised by their low accuracy and reliability under different environmental and manmade conditions [3], and they often require calibration to match the accuracy of traditional systems [4].

PA sensors operate using an optical sensing principle, specifically, laser-based light scattering. Each sensor contains a low-cost laser particle counter that detects airborne particulate matter by illuminating these particles with a laser and measuring the intensity of the scattered light [5]. Specifically, PA sensors utilize the PMS*003 series laser particle sensor, which, unlike many commercial sensors, can measure particulate matter (PM) in three size ranges: PM_1.0_ (particles with a diameter of less than 1.0 μm), PM_2.5_ (less than 2.5 μm) and PM_10_ (less than 10 μm). Their detection range is 0.3–10 µm, with a resolution of 0.3 µm. The performance can also be affected by high humidity, and they record environmental variables such as temperature, humidity, and pressure for further processing. These sensors also include dual laser systems, allowing for cross-validation and improved data quality.

For this study, we focused on PM_2.5_, particulate matter with a diameter of less than 2.5 μm, which can penetrate deep into the respiratory tract and enter the bloodstream, causing health risks, including respiratory, cardiovascular, and neurological diseases [6]. However, the AI/ML approach will not be applicable in instances where particle sizes are smaller than 300 nm, as this is a physical limitation of existing sensor technologies. Monitoring PM_2.5_ is necessary for assessing exposure and implementing strategies to mitigate public health impacts. Large amounts of PA data are available, and previous studies have proved the potential of machine learning (ML) approaches to improve accuracy [7]. Calibration is one of the first investigated methods that has been using AI/ML to correct inherent sensor biases and ensure the comparability of data across different sensors and environments. However, the challenge with PM_2.5_ calibration lies in its sensitivity to ambient environmental changes, such as relative humidity and temperature, which can negatively impact sensor performance and accuracy [8]. Although previous studies (such as [4,9]) have explored ML models for sensor calibration, none has yet provided a comprehensive comparison across as many models and environment variables as this study proposes, nor covered entire sensor networks over a large geographic area.

In this study, fine-tuning refers to the process of making adjustments to ensure correct readings from devices, while calibration is defined as the preliminary step of establishing the relationship between a measured value and a device’s indicated value. Calibration is a preliminary step before tuning. Although we focused on fine-tuning to achieve accurate PM_2.5_ measurements, we refer to this process as calibration, in alignment with the existing literature.

This study focused on the Los Angeles region, selected due to its high density of PA sensors compared to other regions. This area encompasses a mixture of urban, industrial, and residential zones, providing a diverse range of air quality conditions for a more comprehensive evaluation of sensor performance across different environmental settings.

In general, this study sought to bridge the gap between the affordability of LCS and the precision required for scientific and regulatory purposes. Our objective was to systematically evaluate AI/ML models and software packages to identify the most effective model and software package for improving the accuracy of low-cost sensor (LCS) measurements. Each of the AI/ML models was tested to identify the optimal model and package. In total, 64 pairs of Purple Air (LCS) and EPA sensors were used in this study, with the validated EPA measurements as ground truth. Eleven regression models were systematically considered across four Python-based software packages: XGBoost, Scikit-learn, TensorFlow, and PyTorch, as well as a fifth R-based IDE, RStudio. The models in this study included Decision Tree Regressor (DTR), Random Forest (RF), K-Nearest Neighbor, XGBRegressor, Support Vector Regression (SVR), Simple Neural Network (SNN), Deep Neural Network (DNN), Long Short-Term Memory (LSTM) neural network, Recurrent Neural Network (RNN), Ordinary Least Square Regression (OLS), and Least Absolute Shrinkage and Selection Operator (Lasso) regression. The details are provided in the following five sections: Section 2 reviews existing calibration methods conducted using both traditional calibration techniques (field and laboratory methods) and recent advancements involving empirical and geophysical ML models. Section 3 introduces the study area, data, and pre-processing for the PA sensors. Section 4 reports the experimental results. Section 5 presents the results and Section 6 discusses the reasons for model performance differences, comparisons with existing studies, limitations, and future research directions.

## 2. Literature Review

### 2.1. AQ Calibration

AQ calibration has advanced in recent years to address inherent biases and uncertainties from electronics, installation, and configurations, serving as a crucial process to align readings with established reference standards and uphold data validity [10]. Several calibration methods stand out for their efficacy and application diversity. Field calibration, which involves the direct comparison of sensor data with reference-grade instruments in the environment, is used to ensure in situ sensor accuracy [11]. Additionally, laboratory calibration techniques, which subject sensors to controlled conditions and known concentrations of pollutants, allow for the meticulous adjustment of sensor responses before their deployment in the field [12,13]. Calibration techniques also vary by pollutants and sensors. Metal Oxide Semiconductor (MOS) sensors, used for detecting NO_2_ (nitrogen dioxide), O_3_ (ozone), SO_2_ (sulfur dioxide), CO (carbon monoxide), and CO_2_ (carbon dioxide), undergo calibration to correlate electrical conductivity changes with specific target gas concentrations, ensuring accurate readings [14]. Electrochemical (EC) sensors for CO, NO_2_, and SO_2_ monitoring undergo calibration via controlled oxidation–reduction reactions, linking measured currents to gas concentrations for accurate field readings [15]. Non-Dispersive Infrared (NDIR) sensors for CO_2_ measurement require calibration that accounts for spectral variations. This involves exposing the sensor to a range of CO_2_ concentrations and analyzing infrared light absorption patterns to ensure accurate CO_2_ detection [16]. Satellite sensors were introduced to extend AQ observations to regional and global scales and relevant calibration methods were developed for, for example, post-launch atmospheric effects calibration [17,18,19].

Calibrating AQ sensors is essential for maintaining data integrity, particularly when reconciling the lower precision of emerging LCSs with the established accuracy of reference-grade instruments. While field calibration directly aligns sensors with real-world conditions, laboratory techniques refine sensor accuracy under controlled parameters. Despite these advances, the challenge remains to develop calibration methodologies that can navigate the complex interplay of sensor responses with dynamic environmental factors—a focus area that warrants a systematic investigation to enhance AQ monitoring frameworks.

### 2.2. Calibration of LCSs for PM Measurement

LCSs are revolutionizing AQ monitoring by making it more accessible and participatory [20], especially for under-served communities, to collect vital PM data [21]. This grassroots approach offers a richer, more localized view of AQ than is possible with sparser, traditional monitoring networks [22]. However, the accuracy of LCSs is low due to various factors, including environmental influences [23,24] and inherent limitations of the sensors themselves [25].

Most LCSs use light scattering to count particle numbers [26], which is sensitive to fluctuations in temperature, pressure, and humidity [27,28]. Although many LCSs come with built-in mechanisms to track environmental factors and are encased in protective shells to lessen weather impacts, data accuracy is still significantly compromised under extreme weather conditions or values [29,30]. Moreover, different LCS types and their corresponding data interpretation models introduce biases related to the sensor’s location, varying humidity levels, and the hygroscopic growth of aerosol particles [30]. For example, at relative humidity (RH) levels below 100%, hygroscopic PM_2.5_ particles, such as sodium chloride (NaCl), can absorb moisture from the air, leading to an increase in particle size and a change in their optical properties. These alterations can significantly affect the light scattering process, which is central to the operation of LCSs [31,32].

These investigations emphasize rigorous calibration and correction methods to counteract the influence of environmental and other factors, thereby enhancing data reliability in diverse environmental conditions [13,27,33]. While field calibration is essential for aligning LCS readings with standard measurements—requiring placement alongside reference monitors for measurement refinement [20,32]—the development and application of robust and well-performing calibration models are paramount. Such models, when implemented across the sensor network, significantly enhance data consistency and reliability, thereby augmenting the overall efficacy of AQ monitoring efforts.

### 2.3. Models to Calibrate LCSs

There are two types of models for calibrating LCS data to enhance accuracy in AQ monitoring—physics-based models and empirical models. The physics-based model employs fundamental physical principles, such as the κ-Köhler theory and Mie theory, to accurately correlate the sensor’s light scattering measurements [34]. For example, ref. [35] applied κ-Köhler and Mie theories to a low-cost PM sensor, significantly enhancing the accuracy, with coefficient of determination (R^2^) values of up to 0.91 and lower Root Mean Square Error (RMSE) and Mean Absolute Error (MAE) values. Ref. [36] showcased a physics-based calibration approach for PA sensors, which, when aligned with Beta Attenuation Monitor (BAM) standards, exhibited high consistency, with correlations above 0.9, alongside an MAE of 3–4 µg/m^3^.

Empirical models leverage the availability of large amounts of observed data to establish a statistical relationship between sensor readings and reference measurements, often incorporating environmental variables to enhance the accuracy and reliability of LCSs [20,37]. The empirical calibration models commonly assume a correlation between the data from LCSs and high-quality reference-grade measurements. For example, ref. [25] reported a linear calibration model for PM_2.5_, evidencing an enhanced fit with an R^2^ of 0.86 under dry conditions and 0.75 under humid conditions compared to reference measurements. Refs. [10,38,39] emphasized the importance of including environmental variables, notably relative humidity, which affects particle count and sensor outputs. To address these challenges, non-linear and ML models have been utilized for better alignment with high-quality reference instruments [40,41].

Given the critical role of environmental variables, it is important to consider the strong correlation between RH and temperature, which can significantly influence the development of calibration models by introducing multicollinearity, potentially leading to biased predictions [24]. In linear models, for instance, collinearity can distort regression coefficients, making it difficult to assess the true impact of each variable [41,42]. Modern machine learning (ML) models, such as random forests, address this by incorporating the interrelationships between these variables into their algorithms, allowing them to account for correlations when determining variable importance [42,43].

The current literature presents a wealth of individual cases examining the calibration of LCSs with quite limited settings, e.g., limited numbers of sensors and input values. It can also be found that there is no collective, comparative, and systematic study that encompasses diverse calibration models and software packages. This gap is critical because PM_2.5_ measurements from LCSs can vary significantly due to inherent differences in sensing technology, geographical regions, and environmental conditions. Without a systematic approach considering these factors, calibrations may not effectively address these variations, leading to inaccurate data and hindering our ability to fully understand AQ variations. Therefore, our objective was to conduct an in-depth systematic study of AI/ML models and packages to identify the most accurate combinations and improve the accuracy of LCS measurements using AI/ML approaches. We utilized five popular software packages and 11 ML models, mentioned at the end of Section 1, to conduct an in-depth, systematic analysis for a more detailed and nuanced understanding of LCS behaviors and their alignment with standard observations.

## 3. Data and Methodology

### 3.1. Training Data Preparation

**Figure 1:**
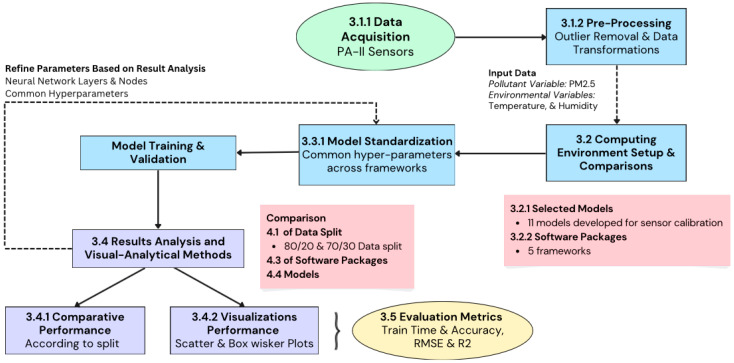
The research workflow includes five major steps including data processing, computing, model setup, experiments, and analyses. Each step is detailed in the subsections.

Figure 1 illustrates a detailed workflow of our study, starting from data acquisition and pre-processing, through model standardization and training, to results analysis and visualization. After step 3.4, where metrics were produced by the different software packages across computing environments, visualizations were produced using RStudio’s ggplot2 library. These visualizations enabled a thorough comparative performance analysis according to different splits, software packages, and models.

#### 3.1.1. Data Acquisition

We downloaded the PA-II sensor data (PMS-5003) and relevant pressure, temperature, and humidity data (AQMD, 2016). Data were kept on two database tables: a sensor table and a reading table. We also utilized data from the U.S. Environmental Protection Agency (EPA) as a benchmark to ensure the accuracy and reliability of our study [44]. EPA sensors utilize Federal Reference Methods (FRMs) and Federal Equivalent Methods (FEMs) [45] to ensure accurate and reliable measurements of PM. These methods involve stringent quality assurance and control (QA/QC) protocols, gravimetric analysis, regular calibration, and strict adherence to regulatory standards. The EPA’s monitoring stations continuously collect data that undergo rigorous validation before being used to determine compliance with National Ambient Air Quality Standards (NAAQS) [45]. Given these processes, EPA data are often considered the gold standard, making them an essential reference for comparing non-regulated sensors.

**Figure 2:**
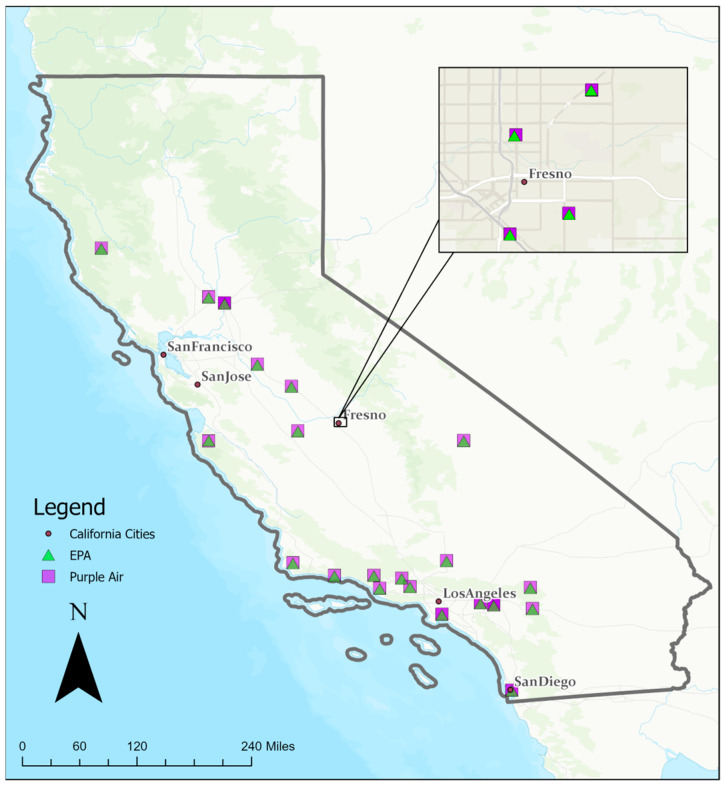
Map of collocated California sensor pairs.

Data were kept on two database tables: a sensor table and a reading table. The sensor table included metadata including hardware component information, unique sensor ID, geographic location, and indoor/outdoor placement. The reading table stored continuous time series data for each sensor, with sensor IDs as primary keys to link the records in both tables. The date attribute was set in UTC for all measurements including pollutant and environmental variables. The table included two types of PM variables: ATM (where Calibration Factor = Atmosphere) and CF_1 (where Calibration Factor = 1) for three target pollutants: PM_1.0_, PM_2.5_, and PM_10_. CF_1 used the “average particle density” for indoor PM and CF_ATM used the “average particle density” for outdoor PM. The PA sensors utilized in this study were outdoor sensors. We identified 64 sensor pairs across California for a total of 876,831 data entries from 10 July 2017 to 1 September 2022 (Figure 2). These 64 sensor pairs consisted of 64 unique PA sensors and 25 unique EPA sensors; these sensors were paired based on their proximity to one another. These sensor pairs were mostly within 10 m of one another, and the furthest distance was below 100 m.

#### 3.1.2. Data Pre-Processing

Data preprocessing utilized a threshold of >0.7 Pearson correlation coefficient between “epa_pm25” and “pm25_cf_1” to ensure a strong linear relationship. Next, data were aggregated from a two-minute temporal resolution into an hourly resolution and adjusted for local time zones. Sensor malfunctions such as readings exceeding 500 were removed, as were data records with missing information from either the “pm25_cf_1_a” or “pm25_cf_1_b” columns. Additionally, readings with a zero 5 h moving standard deviation in either channel were removed as this indicated potential sensor issues. Finally, we applied a dual-channel agreement criterion grouped by year and month. The data were then reduced to the following columns: “datetime”, “pm25_cf_1”, “humidity”, and “temperature”, which composed the training data.

For the LSTM and RNN models, we created sequences using the previous 23 h of data for each sensor. For all models, we then split the data into training and testing sets and scaled them using a standard scaler in a random fashion.

### 3.2. Computing Environmental Setups and Comparisons

For this study, we tested 11 ML models across 5 different packages: Scikit (1.3.2), XGBoost (2.0.2), Pytorch (1.13.1), TensorFlow (2.13), and RStudio (2023.09.1 running R4.3.2). These packages were chosen for their respective strengths and popularity in the academic community. We utilized the same training data and models across each package, all on a consistent machine configuration featuring Microsoft Windows 11 Enterprise OS, a 13th gen Intel(R) Core (TM) i7-13700 at 2100 MHz, 16 cores, 24 logical processors, and 32 GB of RAM. After a thorough analysis and literature review of models supported by each package, we selected 11 AI/ML models suitable for the calibration task (Table 1).

#### 3.2.1. Selected Models

The 11 regression and ML models (detailed in Appendix A as DTR, RF, kNN, XGBRegressor, SVR, SNN, DNN, LSTM, RNN, OLS, and Lasso) that supported calibration required two types of independent and target variables represented by X and Y, respectively, with the goal of mapping a function such that y = f(x_n) + ε, where ε is the degree of error and x_n encapsulates more than one independent variable (e.g., temperature and relative humidity). The strong correlation between temperature and relative humidity could introduce multicollinearity into the model, which could complicate the estimation of the individual effects of these variables on the target outcome. In traditional regression models, this multicollinearity can lead to the inflated variance of the coefficient estimates, potentially resulting in less reliable predictions. However, in the context of the 11 ML models, they were designed to handle such correlations more robustly, either by regularization techniques, e.g., Lasso regression, or by leveraging the complex interrelationships among the variables, e.g., RF, XGBoost, thus minimizing the adverse effects of multicollinearity on the calibration process. We applied regression algorithms to develop similar functions that described the impact of the input variables (measurements) from in situ PA sensors against the measurements aligning with the EPA readings.

### 3.3. Software Packages

The five packages included XGBoost, Scikit-Learn, TensorFlow, PyTorch, and RStudio, as detailed in Appendix B.

Each of the five packages offers unique strengths and limitations, and each available model in the five packages was used to identify the best-suited model and package for PM_2.5_ calibration. For our systematic study, the packages, models, and training data were tested to obtain comprehensive analyses. The training process was repeated 10 times for each experiment, and we calculated an average value for the performance metrics (R^2^ and RMSE).

Note: How PyTorch and TensorFlow implemented the OLS Model:

PyTorch and TensorFlow employed an SNN to define the OLS regression model. Neural networks process simple sequences of feed-forward layers [46]. However, these two packages differ in how they define the model and add layers. TensorFlow utilizes a sequential API for model definition, while PyTorch uses a class-based approach [47]. Moreover, in TensorFlow, the computational graph is a static computation graph, while PyTorch uses a dynamic computation graph. The performance gap between the two packages may stem from differences in these computation graph implementations. Nodes represent the neutral network layers, while edges carry the data as tensors [48].

#### Model Configuration Standardization Across Packages

For comparability across packages and models, we standardize model configuration and hyperparameters for each model. For neural network architectures, we standardized the number and type of layers and the number of nodes of each layer across packages compatible with each model. For several models, such as XGBoost and DTRs, it was not possible to completely standardize models across packages because each software and associated package utilized different hyperparameters. In these cases, we used default hyperparameters unique to each package and ensured that common hyperparameter values across packages were the same. Each model can have dozens of hyper-parameters, we only include those hyper-parameters which were common across packages (Table 2).

The model configuration standardization used the hyperparameters displayed in Table 2 and included the following four aspects:

- Model Configuration: Each model was configured using the default hyperparameter settings provided in their documentation to ensure that they were consistent across all packages. In cases where there were no default hyperparameter settings listed, we set hyperparameters to be equal to the most frequently occurring hyperparameter for that setting. This approach ensured consistency across implementations while maintaining the integrity of each model’s intended configuration. For neural network models, the number and type of layers, the number of nodes per layer, activation functions, and optimization methods were standardized. For tree-based models and regressions, parameters like tree depth, learning rates, and regularization terms were kept consistent.
- Data Preparation: Data input into each model were prepared using a standard preprocessing pipeline. This involved scaling features, handling missing data, and transforming temporal data into sequences for time series models like LSTM models.
- Training and Test Splits: The data were split into training and testing sets, using both 80/20 and 70/30 splits to ensure consistency across all experiments.
- Computation Environment: All models were trained on a consistent hardware setup to eliminate variations in computing resources.

### 3.4. Results and Visual–Analytical Methods

#### 3.4.1. Comparative Performance Across Models and Packages

We evaluated each model and package based on two key criteria: time to train and accuracy (RMSE and R^2^). Averaging the performance metrics (R^2^ and RMSE) from 10 runs of each model provided insight into which packages delivered higher accuracy and reliability. This allowed us to consider both the ability of a particular configuration to accurately calibrate LCS and their suitability for various applications. We identified which models offered the best trade-off between training time and predictive accuracy.

We also considered the influence of the package (e.g., RStudio) and model (e.g., LSTM) on results. These factors were highly intertwined, and the performance of a particular setup depended on both the package and ML model. As such, we took a two-pronged approach to analyze the results. First, we assessed the average performance of a model across all packages or a package across all models. Then, we assessed the effect that package choice had by comparing each model’s relative performance across all packages. By considering each model individually and comparing the difference in results when training in one package or another, we could better analyze the influence of package and model choice.

#### 3.4.2. Visual–Analytical Methods

To succinctly convey our findings, we employed several visual–analytical methods using the “ggplot2” package in RStudio:

- Line and bar graphs were used to plot the performance metrics for the 70/30 and 80/20 splits across all models and packages, illustrating the differences and their consistency.
- A series of box and whisker plots were used to depict the range and distribution of performance scores within each package. This visualization highlighted the internal variability and helped identify packages that generally over- or underperformed.
- Model-specific performance was displayed using both box and whisker plots and point charts. The box plots provided a clear view of variability within each model category, while the point charts detailed how model performance correlated with package choice, effectively illustrating package compatibility and model robustness.

These visual analytics together with the model evaluations can help to refine the selection process for future modeling efforts and ensure that the most effective model/package is chosen for AQ calibration tasks.

### 3.5. Evaluation Metrics

This study utilized the evaluation metrics Root Mean Square Error (*RMSE*) and Coefficient of Determination (*R*^2^) to evaluate the fit of the PM_2.5_ calibration models against the EPA data used as a benchmark.

The *RMSE* was calculated using the formula

$$RMSE=\sqrt{\frac{\sum_{i=1}^{n}\left(y_{i}-{\hat{y}}_{i}\right)}{n}}$$

where *y_i_* represents the actual PM_2.5_ values from the EPA data, ŷ*_i_* denotes the predicted calibrated PM_2.5_ values from the model, and *n* is the number of spatiotemporal data points. This metric measured the average magnitude of the errors between the model’s predictions and the actual benchmark EPA data. A lower *RMSE* value indicated a model with higher accuracy, reflecting a closer fit to the benchmark.

The Coefficient of Determination, denoted as *R*^2^, was given by

$$R^{2}=1-\frac{RSS}{TSS}\;$$

In this formula, *RSS* is the sum of the squares of residuals—the difference between the actual and predicted values—and *TSS* is the total sum of the squares—the difference between the actual values and their mean value. *R*^2^ represents the proportion of variance in the observed EPA PM_2.5_ levels that was predictable from the models. An *R*^2^ value close to 1 suggested that the model had a high degree of explanatory power, aligning well with the variability observed in the EPA dataset.

For a comprehensive understanding of the model’s performance, the RMSE and R^2^ were obtained. The RMSE provided a direct measure of prediction accuracy, while the R^2^ offered insight into how well the model captured the overall variance in the EPA dataset. Together, these metrics were crucial for validating the effectiveness of the calibrated PM_2.5_ models in replicating the benchmark data. The RMSE was more resistant to systematic adjustment errors than R^2^ and, as such, was used as the primary metric.

Furthermore, we investigated the training time for different models to identify “sweet spots”—models that were exceptionally accurate compared to their training time. This analysis is crucial for optimizing model selection in practical scenarios where both time and accuracy are critical constraints.

## 4. Experiments and Results

To obtain a comprehensive result, we implemented a series of experiments to compare the impacts of training/testing data splits, packages, and ML models on accuracy and computing time.

### 4.1. Training and Testing Data Splits

**Figure 3:**
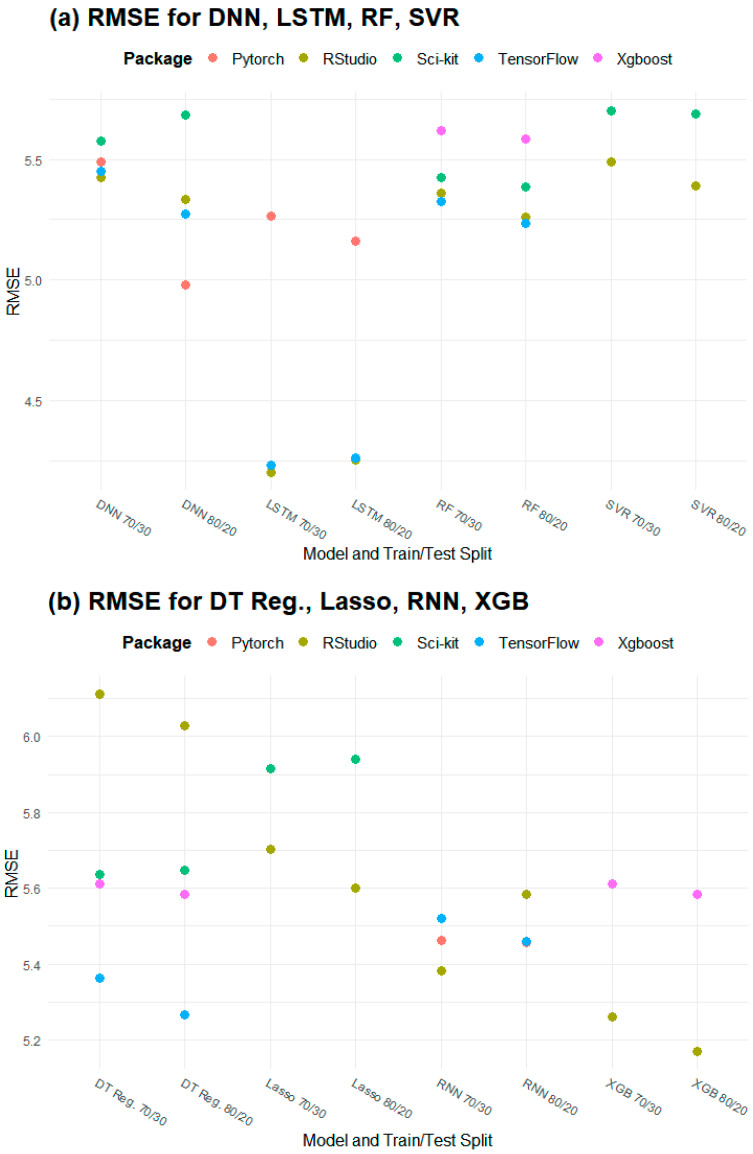

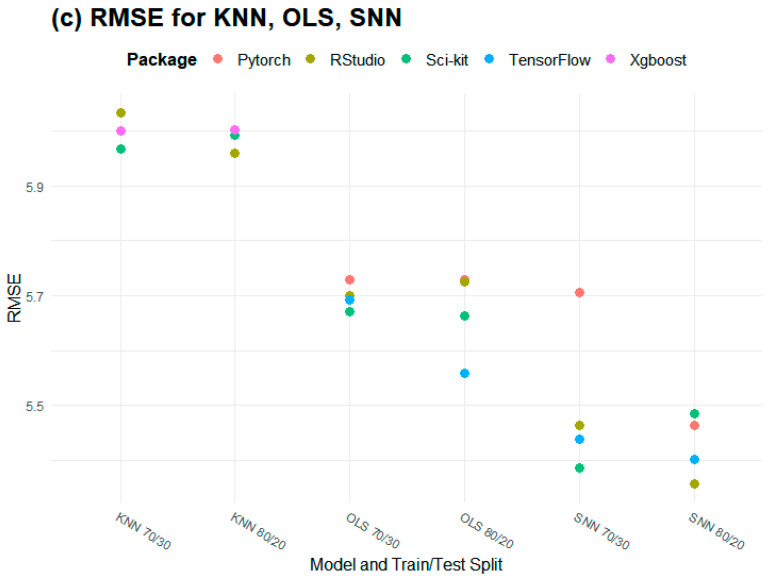
RMSE differences according to training/testing data split (in µg/m^3^): (**a**) DNN, LSTM, RF, SVR; (**b**) DT Reg, Lasso, RNN, XGB; (**c**) RNN, OLS, SNN.

The popular training data splits of 80/20 and 70/30 were examined. The choice of an 80/20 vs. a 70/30 split was found to have minimal impact across models and packages where splits were random, while there was a 2.2% difference in R^2^ performance and a 3% difference in RMSE performance in the 80/20 vs. the 70/30 LSTM model where splits were sequential. The mean difference between the two splits in RMSE across all models and packages was 0.051 µg/m^3^ and the mean difference in R^2^ was 0.00381; there was a mean percent difference of 1.55% for RMSE and a mean percent difference of 0.745% for R^2^ across all packages and models (Figure 3).

The largest difference between the 70/30 and 80/20 splits in terms of RMSE was for DNNs in PyTorch, with an absolute difference of 0.51 µg/m^3^; the largest difference in terms of R^2^ was 0.020 for SNN in PyTorch. This translated to percent differences of 9.75% and 2.83%, respectively. Of the 35 model/package combinations tested, 29 had a difference below 2% for RMSE and 33 had a percent difference below 2% for R^2^ (Figure 3).

While the differences between splits were minimal (Figure 3), the 80/20 split (mean R^2^ = 0.750, mean RMSE = 5.46 µg/m^3^) slightly outperformed the 70/30 split (mean R^2^ = 0.746, mean RMSE = 5.51 µg/m^3^) on average. Therefore, we used the 80/20 split to compare the packages and models.

### 4.2. Software Package Comparison

**Figure 4:**
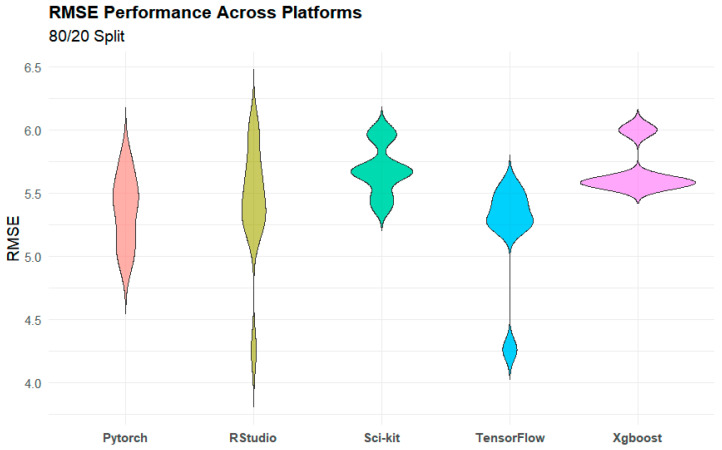
Performance across packages for RMSE (unit µg/m^3^).

When considering the performance of all models (Figure 4), TensorFlow (mean R^2^ = 0.773) and RStudio (mean R^2^ = 0.756) outperformed the other packages, none of which had an average R^2^ above 0.736. This success was driven in part by the strong performance of LSTM in these packages. Apart from RNN, the top-performing packages across models in terms of maximum R^2^ were RStudio and TensorFlow. PyTorch produced the best model for RNN (R^2^ = 0.7658), slightly edging out TensorFlow (R^2^ = 0.7657). Conversely, XGBoost and Scikit-Learn did not produce the best results for any of the models tested. TensorFlow emerged as a package particularly well-suited to the calibration task because every model that TensorFlow supported produced either the best or second-best R^2^ (Figure 3).

However, it is important to note that in cases where a model was compatible with several different packages, the difference in performance between the best and second-best packages was negligible. When considering only models that were compatible with three or more packages, the average percent difference in R^2^ between the top-performing package and the worst-performing package across all models was 6.09%. However, the percentage difference between the top-performing packages and the second-best packages was only 0.96%.

This suggests that while packages did have a significant effect on performance, for each model, multiple potential packages could produce effective results.

Because not every model was available for every package, comparing overall performance did not fully capture the variation between packages. By considering the relative performance of individual models between packages, we could better elucidate which model and package combination was best suited to the calibration task. While many of the models were consistent across packages, there were some notable outliers. OLS regression displayed the largest difference in performance across packages in terms of R^2^, with a percentage difference of 10.06% between the best-performing package (TensorFlow) and the worst-performing package (PyTorch). This difference could be attributed to the different methods that these packages used to calculate linear regression, as discussed in Section 3.2. DTR, LSTM, and SNN all saw a percentage absolute difference of 9% to 10% for R^2^ between the best-performing and worst-performing models. Other models had a percentage absolute difference between 1% and 5% across packages (Table 3).

The effect of package choice was even more pronounced when considering the RMSE. For example, the absolute percent difference between LSTM when training in the worst-performing package, PyTorch, and in the best-performing package, TensorFlow, was 19.3% (Table 4). In certain cases, like LSTM, the selection of packages could have a significant effect on performance, even when the same model was selected. The best performing model is highlighted in bold in Table 4.

While LSTM produced the best results in all packages that supported the model, it was significantly more accurate when trained in RStudio and TensorFlow than in PyTorch (Table 3 and Table 4). It is unsurprising that RStudio and TensorFlow exhibited notably similar performances because LSTM in RStudio was powered by TensorFlow.

The time and performance differences between PyTorch and TensorFlow may have been the result of the different ways that the two packages implemented the models. TensorFlow incorporates parameters within the model compilation process through Keras. In contrast, in PyTorch parameters are instantiated as variables and incorporated into custom training loops, as opposed to the more streamlined .fit() method utilized in TensorFlow [49]. Furthermore, PyTorch employs a dynamic computation graph for the seamless tracking of operations, while the TensorFlow static computational graph requires explicit directives [47]. PyTorch leverages an automatic differentiation engine to compute derivatives and gradients of computations. Moreover, PyTorch’s DataLoader class offers a way to load and preprocess data, thus reducing the time required for data loading.

### 4.3. Model Comparison

Each model’s performance was determined by the model itself and the supporting package. However, the model chosen had a greater overall effect on accuracy than which package was selected. Certain models generally outperformed or underperformed regardless of package.

The top-performing model by average R^2^ and RMSE across software packages was LSTM, which outperformed all other models by a large margin (R^2^ = 0.832, RMSE = 4.55 µg/m^3^) (Table 3 and Table 4). LSTM and RNN are a type of neural network specifically designed for time series modeling and incorporate past data to support predictions of future sensor values [50].

**Figure 5:**
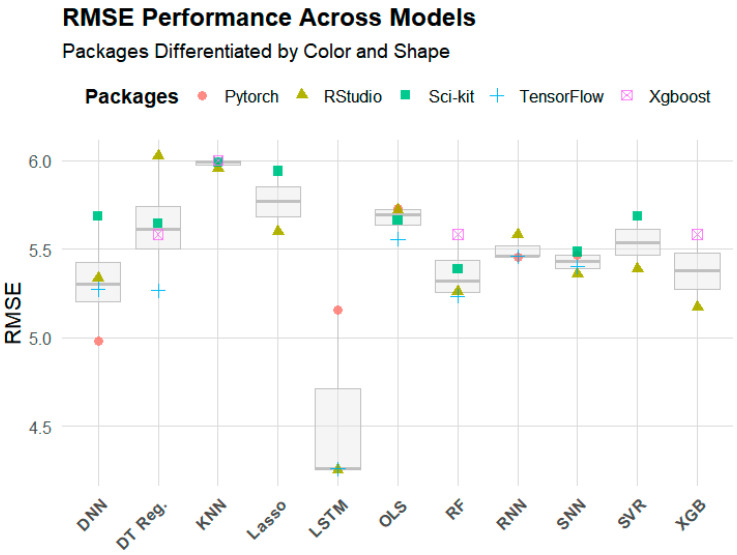
RMSE values (µg/m^3^) across models and packages.

Compared to LSTM, all other models significantly underperformed. Variation in performance among the remaining models was relatively minor (Figure 5). In fact, the gap in mean R^2^ (0.07) between LSTM and the second-best model, RF, was larger than the gap between RF to the worst-performing model, KNN (0.06) (Table 3). The same pattern held true for the RMSE. The gap between LSTM and the second-best model in terms of mean RMSE, DNN, was 0.76 µg/m^3^. The difference between DNN and KNN was 0.66 µg/m^3^.

Table 4 and Table 5 summarize the percentage difference in R^2^ and RMSE between models when considering the best-performing packages for each model. The percentage difference between each model’s best performer compared to the median performer, SNN, and the worst performer, KNN, is included. While LSTM outperformed the median by 11.48% in terms of R^2^, none of the other models was more than 7% different from the median. In fact, 8 of the 11 models had an R^2^ within 3% of the median performance. When comparing models to the worst performer, the same trend was evident. While LSTM had an R^2^ 18.46% higher than the worst performer, no other model outperformed the minimum by more than 9.1%. The same pattern held true for the RMSE, although there was slightly more variance between the models. LSTM again was by far the best performer, with a 23% lower RMSE value than the median model (DNN by this metric). All other models were within 10.6% of the median, and 8 of the 11 models were within 5% of the median value (Table 6).

**Figure 6:**
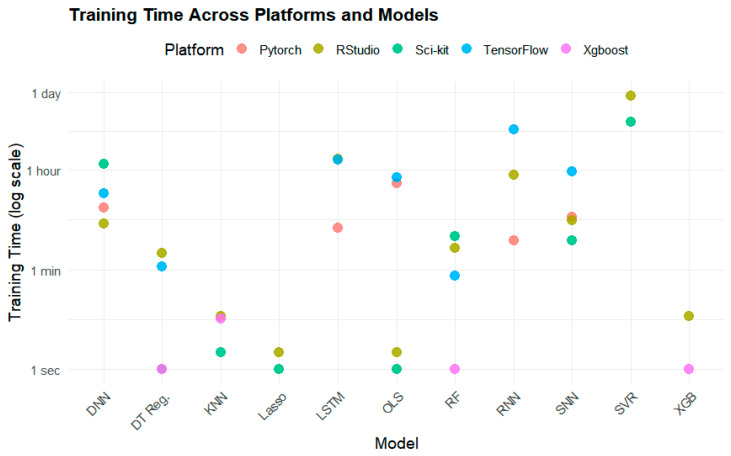
Time to train by model and package in the 80/20 split. Note that the y-axis specifies time non-linearly.

While these models displayed a relatively minor difference in performance in terms of R^2^ and RMSE, their training time was vastly different. Figure 6 demonstrates the elapsed training time across these models. For example, XGBoost took only 5 s to train on average, while SVR took 13 h, 45 min, and 17 s to train (Table 7).

**Figure 7:**
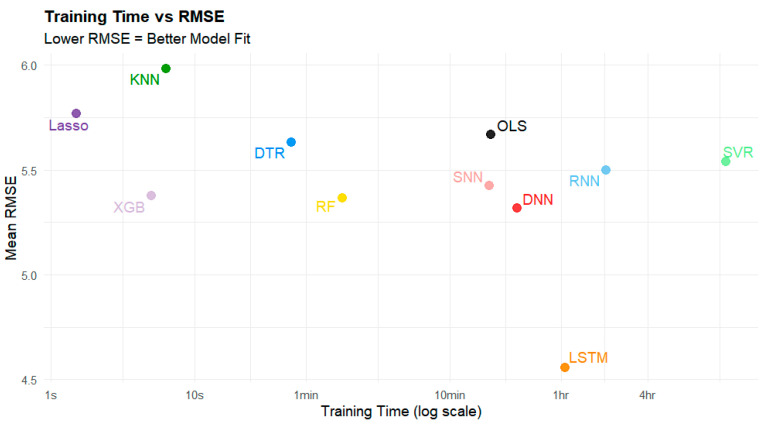
Time to train vs. RMSE (µg/m^3^) in the 80/20 split. Note that the x-axis is non-linear.

While LSTM produced a high R^2^ value, it took significantly longer to train than most other models (Figure 6, Table 7, where NA means not applicable). The fastest models to train were DTR, XGBoost, RF, KNN, and Lasso, all of which took less than two minutes to train. Among these models, XGBoost (0.7612, 5.377 µg/m^3^) and RF (0.7632, 5.366 µg/m^3^) performed the best in terms of R^2^ and RMSE (Figure 7).

These results indicate that the LSTM model in TensorFlow and RStudio provided the highest accuracy, making it suitable for real-time AQ monitoring applications, where high precision is crucial.

## 5. Discussion

This section elaborates on why the 11 models performed differently, how our results compare to the latest relevant research, the applicability of our research to other sensors, and the limitations of this study.

### 5.1. Model Performances and Structures

Purple Air and EPA’s PM_2.5_ datasets are inherently point-based, hourly time series that require models to account for time series handling. These PM_2.5_ readings are influenced by complex and dynamic relationships with meteorological factors like temperature and relative humidity, so these need to be considered as variables in addition to raw PM_2.5_ data when building a model. As evidenced in Table 8, models like RF, DT, and XGB, while effective for multivariable inputs and feature interaction, treat data points independently. This makes them less suitable for time series tasks without significant feature engineering [51]. Meanwhile, models such as KNN and SVR lack the capability of capturing time series patterns, limiting their effectiveness for time series-based calibration [52]. However, LSTM models excel in handling time series data by using hidden and cell states to learn temporal patterns across time steps [53] and capture these nonlinear and interdependent relationships between the input variables and the output with their combined influences through their ability to utilize time series data. Forget and memory gates enable LSTM models to smooth irregular patterns in PM_2.5_ data, reducing the impact of noise and sensor errors commonly found in environmental datasets [54]. Unlike traditional RNNs, which suffer from the vanishing/exploding gradient problem, LSTM’s architecture is better suited to this task [54,55]. This design allows LSTM models to retain long-term dependencies, which are essential in understanding the temporal patterns in PM_2.5_ data [56,57]. In conclusion, LSTM’s dual hidden state allows it to maintain short- and long-term dependencies across time series data, capture nonlinear influences between PM_2.5_ and meteorological variables without manual feature engineering, and smooth irregular patterns in data through memory gates.

### 5.2. Comparison to Existing Studies

We compared our study with all PM_2.5_ calibrations using AI/ML techniques (Table 9) and a number of factors: sensor count, sensor quality and performance, comprehensive model evaluation, and sensor type and calibration. A key factor influencing model performance was the number of sensors used. This study utilized 64 Purple Air sensors, a significantly larger dataset compared to previous works, which often relied on fewer sensors (e.g., 1–9). The use of a larger sensor network introduced greater data variability, enhancing the model’s ability to capture diverse environmental conditions. The studies by [74,75] demonstrated that calibrating single LCSs using models yielded high R^2^ values. However, their limited sensor count could reduce generalizability across different environmental conditions, as fewer sensors could not fully capture the natural variability present in PM_2.5_ measurements.

Another key factor in this comparison is comprehensive model evaluation. This study systematically evaluated 11 machine learning models across five software packages, including TensorFlow and PyTorch. By incorporating a diverse range of models, from simple linear methods to complex architectures like DNN and LSTM, it offers a more extensive performance assessment than previous studies, which often focused on fewer models within a single framework. While this study evaluated 11 models, ref. [76] used a maximum of three models (ANN, GBDT, RF) in its calibration efforts, demonstrating a more limited exploration of model diversity. Our multi-model, multi-package approach helped identify the best-performing models for the given data while minimizing bias and improving the reliability of the PM_2.5_ calibration results.

The third key factor for comparison is sensor type and quality. The high accuracy reported by [77] was attributed to comprehensive field calibration against industry-grade instruments, ensuring robust validation. Similarly, ref. [78] achieved high accuracy due to the initial high agreement among sensors (R^2^ of 0.89), indicating strong baseline data quality prior to extensive calibration. In contrast, this study utilized 64 Purple Air PMS5003 sensors without direct field calibration but instead applied a much more flexible agreement threshold of R^2^ ≥ 0.70 to ensure data quality while capturing diverse environmental conditions. Additionally, sensor type can influence calibration outcomes, as seen in [50], where the SPS30 sensor paired with a hybrid LSTM calibration model yielded a high R^2^ of 0.93. Despite being a low-cost sensor (LCS), the SPS30 often outperformed other sensors like the PMS5003, likely due to its higher sensitivity and improved particle size differentiation [79].

### 5.3. Applicability of the Results to Other Air Quality Sensors

Table 10 explores the applicability of this methodology to other air quality sensor technologies. This workflow could be applicable to any sensor testing for PM_2.5_; however, the approach may vary depending on each specific sensor’s needs and the technologies that they utilize. This general workflow could be applied to other pollutants and aerosols; however, the results may differ due to the differences in dispersion behavior that occur from pollutant to pollutant.

## 6. Conclusions

This paper reported a systematic investigation of the suitability of five popular software packages and 11 ML models for LCS AQ data calibration. Our investigation revealed that the choice of training/testing split—80/20 vs. 70/30—had minimal impact on the performance across various models and packages. The percentage difference between the model split performance (R^2^) averaged as 0.745% and, therefore, we focused on the 80/20 split for a detailed comparison in subsequent analyses.

In the package comparison, RStudio and TensorFlow were the top performers, particularly excelling with LSTM models. Their performance showed R^2^ scores of 0.8578 and 0.857 and low RMSEs of 4.2518 µg/m^3^ and 4.26 µg/m^3^, respectively. Their strong ability to process high-volume data and capture complex relationships with neural network models such as LSTM was evident. However, while RStudio outperformed TensorFlow by 0.09% for LSTM, TensorFlow typically outperformed RStudio for every other model by 1.7%, averaging an R^2^ of 0.773 in TensorFlow and 0.756 in RStudio.

The choice of packages affected the outcomes when the same models were implemented across different packages. For example, the performance discrepancies in OLS regression across packages underscored the influence of software-specific implementations on model efficacy. When averaging across all models, R^2^ scores varied by 6.09% between the most and least accurate packages.

This study also highlighted the importance of selecting the appropriate combination of model and package based on the specific requirements of the task. While some packages showed a broad range in performance, packages like Scikit-Learn showed less variability, indicating a more consistent handling of the models. While the choice of model generally had a greater impact on performance than the package, the nuances in how each package processed and trained the models could lead to significant variations in both accuracy and efficiency. For example, while LSTMs generally performed well, their implementation in TensorFlow consistently outperformed that in PyTorch. This highlights the differences in how these packages manage computation graphs.

In conclusion, the detailed insights gained from this research advocate for a context-driven approach in the selection of ML packages and models, ensuring that both model and package choices are optimally aligned to the specific needs and constraints of the predictive task. Across all experiments, two optimal approaches emerged. The overall best-performing model in terms of RMSE and R^2^ was clearly LSTM. However, LSTM algorithms are particularly time-intensive to train, each taking over one hour and thirty minutes to train a single model. In addition, preparing sequential training data is a somewhat computationally expensive process. LSTM’s computational demands may make it too slow or expensive to train for certain applications, such as those with large study areas or applications that require model training on the fly. The high computational load of LSTM models is particularly important to consider for in-depth explorations, such as hyperparameter tuning. The hyperparameter tuning of these models can require hundreds of training runs, leading to long calculation times. The results also suggest a second potential approach, indicated by the relatively high performance of tree-boosted models in comparison to their training time. XGBoost in RStudio and RF in TensorFlow both exhibited R^2^ values above 0.77, RMSE values below 5.3 µg/m^3^, and a time to train below one minute. In cases where computational resources are low or models need to be trained quickly on the fly, models such as RF and XGBRegressor may be more applicable than the top-performing time series models.

### 6.1. Limitations

We have presented a systematic calibration study for PM_2.5_ sensors with promising results. There are some limitations that can guide interpretations of the findings and future research. These limitations span the geographic and technological scope of sensor deployment, the pollutant species, computational constraints, and the limited available meteorological variables.

- Sensor Pair Distribution: The current study utilized 64 sensor pairs from California, incorporating data from 25 unique EPA sensors. This limited geographic and technological scope may limit the broader applicability of the models, particularly for nationwide or larger-scale contexts. Further research could be conducted to determine the optimal scope and effectiveness of the trained models across diverse regions.
- Pollutant Species: This calibration study was exclusively focused on PM_2.5_ and did not extend its methodology to other pollutants. The generalizability of the approach to additional pollutants, such as ozone or nitrogen dioxide, could be investigated through similar calibration efforts.
- Sensor Technology: This study was confined to data collected from EPA and Purple Air sensors. While these sensors are widely used, the approach should be repeated when translating to other types of PM_2.5_ sensors or to sensors measuring different pollutants. Future studies should explore the calibration and performance of alternative sensor technologies to enhance this study’s applicability.
- Computational Constraints: The calibration process was conducted using CPU-based processing, which required approximately one month of continuous runtime. This computational limitation suggests that further studies could benefit significantly from leveraging GPU-based processing to reduce runtime [68]. Additionally, adopting containerization technologies such as Docker could streamline setup and configuration, thereby improving efficiency and reproducibility.
- Meteorological Constraints: While this study accounted for the impact of temperature and humidity on sensor calibration, it did not consider other potentially influential meteorological factors, such as wind speed, wind direction, and atmospheric pressure. These features were either found to have marginal impacts in the case of pressure or were unavailable in the dataset such as in cases of wind speed and direction. Further studies with sensors that measure these variables could potentially further improve model accuracy.

### 6.2. Future Work

Though this study is extensive and systematic, four aspects need further investigation to best leverage AI/ML for air quality studies on various pollutants, data analytical components, and further improvements of accuracy for calibration:

- Hyperparameter tuning should be able to further improve accuracy and reduce uncertainty but will require significant computing power and long durations of model training to investigate different combinations. LSTM emerged as the best-performing model in this study. We plan to further explore the application of this model, including detailed hyperparameter tuning/model optimization.
- The incorporation of a broader set of evaluation metrics, including MAPE and additional robustness measures, could provide a more comprehensive assessment of model performance across conditions.
- Different species of air pollutants may have different patterns so a systematic study on each of them might be needed for, e.g., NO_2_ and ozone or methane, within various events such as wildfire and wars [5,79]. In situ sensors offer comprehensive temporal coverage but lack continuous geographic coverage; introducing the satellite retrieval of pollutants could complement air pollution detection.
- Further exploration of other analytics such as data downscaling, upscaling, interoperation, and fusion to best replicate air pollution status is needed for overall air pollutants data integration.
- To better facilitate systematic studies and extensive AI/ML model runs, an adaptable ML toolkit and potential Python package could be developed and packaged to speed up AQ and forecasting research.
- Additionally, future studies should apply this methodology to datasets from various regions with different climates and pollution levels, as geographic location can significantly impact air quality patterns and model performance. This would help to validate the robustness and generalizability of the models under diverse conditions.

## Figures and Tables

**Table 1:** Models supported by each package.

Models\Packages	XGBoost	Scikit-Learn	Tensorflow	Pytorch	RStudio
DTR	✓	✓	✓		✓
RF	✓	✓	✓		✓
KNN	✓	✓			✓
XGBR	✓				✓
SVR		✓			✓
SNN		✓	✓	✓	✓
DNN		✓	✓	✓	✓
RNN			✓	✓	✓
LSTM			✓	✓	✓
OLS		✓	✓	✓	✓
Lasso		✓			✓

**Table 2:** Standardized hyperparameter settings for each package.

DTR	Max depth = 6
RF	mtry = 3, splitrule = variance, min.node.size = 1
KNN	kmax/n_neighbors = 5
XGBR	eta = 0.1, max_depth = 6, n_estimators = 100, gamma = 0, colsample_by_tree = 1, min_child_weight = 1, subsample = 1
SVR	kernel = “radial”, degree = 3
SNN	epochs = 30, batch_size = 32, learning_rate = 0.0001, input_size = 3, output_size = 1, activation = ReLU, hidden_size = 32, optimizer = adam, loss = MSELoss
DNN	epochs = 30, batch_size = 32, layer type = dense, optimizer = “adam”, learning rate = 0.001, hidden_layers = 3, size = (64, 32, 16, 8, 1), activation = reul for layers 1–4, linear activation for layer 5
LSTM	Units1 = 50, units2 = 50, batchsize = 32, epochs = 30, hidden layers = 2, learning rate = 0.001, optimizer = adam
RNN	epochs = 30, batchsize = 32, optimizer = adam, lrate = 0.001, Layer 1: Simple RNN units 50, laye:simple RNN, units 50, Layer 3: dense, units 1
OLS	Defaults selected for RStudio and SciKit; see note in Section 3.2.1 about neural network implementation of linear regression in PyTorch and Tensorflow.

**Table 3:** Package performance by model (R^2^). Best model in bold.

Model	Mean R^2^	Maximum R^2^	Best Package	Minimum R^2^	Worst Package	Difference R^2^	Percent Difference
DTR	0.7385	0.7750	Tensorflow	0.7018	RStudio	0.0732	9.913
DNN	0.7541	0.7646	RStudio	0.742	PyTorch	0.0226	3.011
KNN	0.7054	0.7128	RStudio	0.7016	XGBoost	0.0112	1.584
Lasso	0.7267	0.7422	RStudio	0.7111	Scikit Learn	0.0311	4.28
**LSTM**	**0.8323**	**0.8578**	**RStudio**	**0.78209**	**PyTorch**	**0.0757**	**9.234**
OLS	0.7192	0.7399	Tensorflow	0.66898	PyTorch	0.0709	10.068
RF	0.7632	0.7756	Tensorflow	0.7417	XGBoost	0.0339	4.468
RNN	0.7619	0.7658	PyTorch	0.7543	RStudio	0.0115	1.513
SNN	0.7454	0.7647	RStudio	0.69883	PyTorch	0.0658	9.002
SVR	0.7480	0.7640	RStudio	0.732	Scikit Learn	0.0320	4.287
XGBR	0.7612	0.7807	RStudio	0.7417	XGBoost	0.0390	5.123

**Table 4:** Package performance by model (RMSE). Best model in bold.

Model	Mean RMSE (µg/m^3^)	Minimum RMSE (µg/m^3^)	Best Package	Maximum RMSE (µg/m^3^)	Worst Package	Difference RMSE (µg/m^3^)	Percent Difference
DTR	5.631	5.2659	Tensorflow	6.028	RStudio	0.7621	13.496
DNN	5.319	4.9802	PyTorch	5.6852	Scikit Learn	0.705	13.22
KNN	5.984	5.959	RStudio	6.0018	RStudio	0.0428	0.716
Lasso	5.767	5.6	RStudio	5.9398	Scikit Learn	0.3398	5.889
**LSTM**	**4.557**	**4.2518**	**RStudio**	**5.15965**	**PyTorch**	**0.90785**	**19.292**
OLS	5.669	5.5575	Tensorflow	5.7293	PyTorch	0.1718	3.044
RF	5.366	5.2349	Tensorflow	5.5833	XGBoost	0.3484	6.441
RNN	5.500	5.4578	RStudio	5.584	RStudio	0.1262	2.286
SNN	5.4269	5.3569	RStudio	5.4854	PyTorch	0.1285	2.37
SVR	5.539	5.39	RStudio	5.6871	Scikit Learn	0.2971	5.364
XGBR	5.377	5.17	RStudio	5.5834	XGBoost	0.4134	7.689

**Table 5:** Deviation from median across models (R^2^).

Model	Best-Performing Package	Best R^2^	Percent Difference of R^2^ from Median	Percent Difference of R^2^ from Minimum
LSTM	RStudio	0.8578	11.48	18.46
XGBoost	RStudio	0.7807	2.071	9.093
RF	TensorFlow	0.7756	1.415	8.439
DTR	TensorFlow	0.775	1.338	8.361
RNN	PyTorch	0.7658	0.1437	7.169
SNN	RStudio	0.7647	0	7.025
DNN	RStudio	0.7647	−0.002615	7.023
SVR	RStudio	0.7641	−0.08242	6.943
Lasso	RStudio	0.7422	−2.986	4.041
OLS	TensorFlow	0.7399	−3.297	3.731
KNN	RStudio	0.7128	−7.025	0

**Table 6:** Deviation from median across models (RMSE, µg/m^3^).

Model	Best-Performing Package	Best RMSE (µg/m^3^)	Percent Difference of RMSE from Median	Percent Difference of R^2^ from Minimum
LSTM	RStudio	4.252	−23.00	−33.44
DNN	PyTorch	4.980	−7.288	−17.90
XGBoost	RStudio	5.170	−3.551	−14.18
RF	TensorFlow	5.235	−2.304	−12.94
DTR	TensorFlow	5.266	−1.713	−12.35
SNN	RStudio	5.357	0.000	−10.64
SVR	RStudio	5.390	0.61560	−10.03
RNN	PyTorch	5.458	1.866	−8.780
OLS	TensorFlow	5.558	3.676	−6.973
Lasso	RStudio	5.600	4.437	−6.212
KNN	RStudio	5.959	10.64	0.000

**Table 7:** Time to train (hh:mm:ss) 80/20 split.

Model	PyTorch	RStudio	Sci-kit	TensorFlow	XGBoost	Average
DTR	NA	0:02:00	0:00:01	0:01:07	0:00:01	0:00:47
DNN	0:12:52	0:06:32	1:15:32	0:22:32	NA	0:34:52
KNN	NA	0:00:09	0:00:02	NA	0:00:08	0:00:06
Lasso	NA	0:00:02	0:00:01	NA	NA	0:00:02
LSTM	0:05:29	1:34:48	NA	1:31:12	NA	1:33:00
OLS	0:34:21	0:00:02	0:00:01	0:43:11	NA	0:14:25
RF	NA	0:02:23	0:03:58	0:00:47	0:00:01	0:01:47
RNN	0:03:19	0:47:52	NA	5:15:13	NA	3:01:33
SNN	0:08:45	0:07:34	0:03:19	0:55:51	NA	0:22:15
SVR	NA	20:56:00	7:03:27	NA	NA	13:59:43
XGBoost	NA	0:00:09	NA	NA	0:00:01	0:00:05

**Table 8:** Each model’s capabilities.

Model	Multivariable Regression Capability	Time Series Capability	Points-Based Data Handling
LSTM	Yes—handles multiple input features for time series [58,59]	Yes—captures long-term temporal dependencies [59]	Yes—handle points-based data by processing inputs in a time series as discrete points in sequential order [58]
RNN	Yes—processes multivariable input features [60]	Yes—maintains memory through recurrent layers [61]	Yes—iterates through each point sequentially at each time step [59]
DNN	Yes—processes high-dimensional static data [62]	Partial—needs integration with RNN/LSTM for temporal processing [63]	Yes—while designed for static data, can process appropriately structured points-based data [62]
SNN	Yes—models multivariable inputs after some minor tuning [63]	Partial—can handle short-term temporal dependencies [64]	Yes—treats each input point as an independent observation and processes these through a series of transformations, allowing for predictions on input features [63]
DT	Yes—splits the data based on feature values [65]	Partial—multiple transformations needed with potential for error at [66]	Yes—splits the input space into regions based on individual feature values at specific points [65]
RF	Yes—ensemble model for multivariable input [67]	Partial—static; not designed for sequential data [68]	Yes—process points-based data by aggregating decisions from multiple decision trees [67]
KNN	Yes—works by spatial proximity on multivariable inputs [68]	Partial—does not handle temporal order well but can be used in univariate [68]	Yes—measures distances between data points in feature space, comparing points to their neighbors to make predictions and classifications [69]
XGB	Yes—optimized for multivariable prediction tasks [70]	No—tree-based; lacks sequential processing capabilities [70]	Yes—handles points-based data through gradient-boosted decision trees, splitting data on individual features at specific points [70]
SVR	Yes—handles multivariable regression tasks [71]	Partial—can handle some temporal dependencies if hybridized with other models [71]	Yes—processes points-based data by finding optimal hyperplane to predict outcomes for individual points in feature space [71]
OLS	Yes—linear regression with multivariable inputs [72]	No—static; no sequential capability [72]	Yes—individual points are represented by multivariable input and target; estimates relationships between points and corresponding outputs linearly [72]
Lasso	Yes—regularizes multivariable data for regression [73]	No—not built for temporal sequences [73]	Yes—represents points-based data by regularizing coefficients in linear models for individual input points, treated as discrete input for the regression model [73]

**Table 9:** Summary of calibration methods for LCSs for PM_2.5_ in various study areas using different models and limitations.

Reference	Study Area	Sensor Type/Name	PM_2.5_ Detection Technique	Parameters Used	Model	R^2^	RMSE (µg/m^3^)	Drawbacks
[25]	Shandong Province, China	PMS5003	Laser light scattering	RH, temperature, windspeed (dry conditions)	Linear	0.86	15.02	Two sensor pairs were used
					GAM	0.88	14.09	
				RH, temperature, windspeed (humid conditions)	Linear	0.75	21.54	
					GAM	0.83	15.17	
[74,75]	Mt. Tai, China	SDS019	Laser diffraction	Temperature, RH, wind speed, pressure	MLR	0.82	N/A	Single sensor calibration
[76]	Columbia core-Based Statistical Area (CBSA)	DustTrak™ DRX 8533EP	Light scattering		GAM	0.82	N/A	Single sensor calibration
[77]	Guildford, UK	PMS5003	Light scattering		SVR	0.87	3.39	Field study was conducted before deployment to benchmark PM against high-quality instruments, which is not typically done for low-cost sensors like Purple Air.
[78]	Jinan, China	DS019-TRF	Laser diffraction	RH	ANN	0.90	13.87	
					GBDT	0.91	13.16	
					RF	0.91	13.44	
[75]	Calgary, Canada	PMS5003	Laser scattering	RH, temperature	NN	0.72	3.91	Single sensor calibration
[50]	South Korea	SPS30	Laser scattering		Hybrid LSTM	0.93		Single sensor calibration

**Table 10:** Applicability scope of various air quality sensors.

Sensor	Meteorological Measurements	Air Pollutant	Applicability Scope and Level
EPA AQS (EPA, 2024)	Temperature, Relative Humidity, Wind Speed, Wind Direction, Barometric Pressure, Solar Radiation	PM_2.5_, PM_10_	Yes, this can be directly applied. However, there is no need since EPA completes verification and validation.
		NO_2_, O_3_, SO_2_, CO	The general approach is applicable, but the results may be different because of the different dispersion behavior of pollutants in the air.
Purple Air (Purple Air, 2024)	Temperature, Relative Humidity, Barometric Pressure	PM_2.5_, PM_1.0_, PM_10_	Yes, this can be directly applied. PA is prone to error, so calibration is recommended.
AERONET (Slutsker and Gupta, 2022)	Temperature, Relative Humidity, Barometric Pressure, Wind Speed, Wind Direction, Precipitation	Aerosols	The general approach is applicable, though results may differ due to differences in the dispersion behavior of various pollutants.
Shinyei PPD42NS (AQICN, 2024)	Temperature, Relative Humidity, Airflow	PM_2.5_, PM_10_	Yes, this approach is potentially applicable.
		Dust	Needs further exploration of applicability.
Alphasense OPC N2 (AQMD, 2024a)	Temperature, Relative Humidity	PM_2.5_, PM_1.0_, PM_10_	Yes, this is potentially applicable. Temperature and RH are known to have direct impacts on sensor performance.
Dylos DC1700-PM (AQMD, 2024b)	Temperature, Relative Humidity	PM_2.5_, PM_10_	Yes, this is potentially applicable. Temperature and RH are known to have direct impacts on sensor performance.

## Data Availability

The training data used in this study are openly available on GitHub at https://github.com/stccenter/AQ-Formal-Study/tree/main/Training%20Data, accessible as of 6 February 2025.

## Appendix A. The Machine Learning Models Used in This Study

1. Decision Tree Regressor (DTR) predicts a target value by learning simple decision rules inferred from training data [80]. It splits the training data into increasingly specific subsets based on feature thresholds, with each leaf node in the tree providing a prediction that represents the mean of the values in that segment.
2. Random Forest (RF) is an ensemble learning model that builds multiple decision trees during training and outputs the average prediction of the individual trees [81]. It improves model accuracy and overcomes the overfitting problem of single trees by averaging outputs from multiple deep decision trees, each trained on a random subset of features and samples.
3. K-Nearest Neighbor (KNN) is a non-parametric model used for regression and classification problems [82]. The output is calculated as the average of the values of its k nearest neighbors. KNN works by finding the k closest training examples in the feature space and averaging their values for prediction.
4. XGBRegressor is part of the XGBoost package that performs highly efficient and scalable implementation of a gradient-boosting framework [83]. This model uses a series of decision trees, where each tree corrects errors made by the previous ones, and it includes regularization terms to prevent overfitting.
5. Support Vector Regression (SVR) is an extension of the Support Vector Machine (SVM) [84]. SVR tries to fit an error within a certain threshold and is robust against outliers. It uses kernel functions to handle non-linear relationships:
  (A1) $$z\left(a,w\right)=\left(w\cdot \varphi \left(a\right)\right)$$
  where *w* is the weight vector, *φ*(*a*) is the feature function representing the input variables, and (*w* * *φ*(*a*)) is the dot product targeting the prediction results.
6. Simple Neural Network (SNN) is often a single-layer network with direct connections between inputs and outputs [85]. It can model relationships in data by adjusting the weights of these connections, typically using methods like backpropagation.
7. Deep Neural Network (DNN) composes multiple layers between the input and output layers (known here as hidden layers), which enable the modeling of complex patterns with large datasets [86]. Each layer transforms its input data into a slightly more abstract and composite representation. Further experimentation is needed to identify the balanced layer number and overfitting for the specific use case of sensor calibration.
8. Recurrent Neural Network (RNN) is a class of neural networks where the node connections form a directed graph along a temporal sequence to exhibit dynamic temporal behavior and process sequences of inputs, making it suitable for tasks like time series forecasting [87].

One setup equation for RNN is

(A2) $$\left(x^{i},y^{i}\right)=\left(\left[x^{i<1>},x^{i<2>},\ldots ,\;x^{i<t>},\ldots ,\;x^{i<T^{i}>}\right],\left[y^{i<1>},y^{i<2>},\ldots ,\;y^{i<t>},\ldots ,\;y^{i<T^{i}>}\right]\right)$$

where $x^{i<t>}$ is the input data point (scalar/real valued vector) at time step t for the *i*(th) training/test example, $y^{i<t>}$ is the target (scalar/real valued vector) at time step t for the *i*(th) training/test sample, ${\hat{y}}^{i<t>}$ is the predicted output (scalar/real valued vector) at time step *t* for the *i*(th) training/test example, $a^{i<t>}$ is the hidden state (real valued vector) at time step t for the *i*(th) training/test example, and $W_{ax},\;W_{ya},\;W_{aa}$ are the weight matrices associated with the input, output, and hidden states, respectively. In this study, $x^{i<t>}$ pertains to the input PM_2.5_, relative humidity, and temperature data points at a certain time *t*.

9. Long Short-Term Memory (LSTM) is a type of RNN that consists of three gates in its memory cell: the forget gate, the input gate (the PM_2.5_, temperature, and relative humidity), and the output gate (the calibrated values) [88].

This is later used to build an LSTM structure:

(A3) $$f_{t}=\sigma \left(w_{f}\cdot \left[h_{t}-1,x_{t}\right]+b_{f}\right)$$

(A4) $$i_{t}=\sigma \left(w_{i}\cdot \left[h_{t}-1,x_{t}\right]+b_{i}\right)$$

(A5) $${\bar{c}}_{t}=\tanh\left(w_{c}\cdot \left[h_{t}-1,x_{t}\right]+b_{c}\right)$$

(A6) $$c_{t}=f_{t}\cdot C_{t-1}+i_{t}\cdot {\bar{C}}_{t}$$

(A7) $$O_{t}=\sigma \left(W_{0}\cdot \left[h_{t-1,x_{t}}\right]+b_{0}\right)$$

(A8) $$h_{t}=O_{t}\cdot \tanh\left(c_{t}\right)$$

where *f_t_* = forget gate, o = sigmoid, *w_f_* = weight, *h_t_* − 1 = output of previous block, *x_t_* = input vector, and *b_f_* = bias. The multiplication is performed elementwise, *c_t_* = cell state, *h_t_* = hidden state, and *O_t_* = output gate. The input sequence comprises the PM_2.5_, relative humidity, and temperature values at a given time *t*.

10. Ordinary Least Square (OLS) regression is a method for estimating unknown parameters [89]. OLS chooses the parameters that minimize the sum of the squared differences between the observed dependent variable and those predicted by the linear function.
11. Lasso (Least Absolute Shrinkage and Selection Operator) regression is a type of linear regression that uses shrinkage (minimizing coefficients) [90]. It adds a regularization term to the cost function, which involves the L1 norm of the weights, promoting a sparse model where few weights are non-zero.

## Appendix B. The Five Open-Source Packages

XGBoost, standing for eXtreme Gradient Boosting, is a highly efficient implementation of gradient-boosted decision trees designed for speed and performance [91]. This standalone library excels in handling various types of predictive modeling tasks including regression, classification, and ranking. XGBoost can be used with several data science environments and programming languages including Python, R, and Julia, among others. XGBoost works well to build hybrid models as it integrates smoothly with both Scikit-Learn and TensorFlow via wrappers that allow its algorithms to be tuned and cross-validated in a consistent matter. It also functions well as a standalone model, using functions like XGBRegressor.

Scikit-Learn is a comprehensive library used extensively for data preparation, model training, and evaluation across a spectrum of ML tasks such as classification, regression, and clustering [92]. It supports many algorithms included in this study and other advanced regression and AI/ML algorithms. This package excels due to its ease of use, efficiency, and broad applicability in tackling both simple and complex ML problems.

TensorFlow, coupled with its high-level API Keras, provides a robust environment for designing a diverse array of ML models [93]. It is particularly effective for developing neural network models such as the ones employed in this study. TensorFlow uses “tf.keras” to implement regression models. RF and Lasso are built with extensions like TensorFlow Decision Forests (TF-DF), demonstrating its versatility across both deep learning and traditional ML domains.

PyTorch, known for its flexibility and powerful GPU acceleration, is used in the development of DL models [94]. While it is not traditionally used for simple regression models, it is ideal for constructing complex neural network models. For some regression modeling, external packages or custom implementations are necessary to bridge its capabilities to traditional statistical modeling tasks.

RStudio facilitates ML through its integration with R and Python, offering access to various packages and frameworks [95]. It utilizes the Caret package for training conventional ML models such as RF and XGBoost. For regression models like OLS and Lasso, RStudio leverages native R packages and Python integrations through Reticulate. Advanced DL models including LSTM and RNN are also supported using TensorFlow and Keras, providing a flexible and powerful toolset for both classical and modern ML approaches.
